# Case report: Fistula formation of a urachal cyst 6 years after prostatectomy

**DOI:** 10.1016/j.eucr.2025.103222

**Published:** 2025-09-22

**Authors:** Anton M.F. Kalsbeek, Arnout R. Alberts, Lucia L. Rijstenberg, John van der Hoeven, Tahlita C.M. Zuiverloon

**Affiliations:** a Erasmus Medical Centre, Rotterdam, the Netherlands; b Reinier de Graaf Gasthuis, Delft, the Netherlands

**Keywords:** Urachus, Mucinous cystadenoma, Prostatectomy, Fistula

## Abstract

Urachal cysts can be a diagnostic dilemma, because determining whether the cyst is malignant can be difficult. As a result, the best treatment is not always obvious. Urachal malignancies account for less than 1 % of total bladder cancer cases thus in most cases urachal cysts are benign, and a watchful waiting approach is justified. However, in cases with symptoms or a suspicion of malignancy, there is an indication for surgical resection. Here, we discuss the management of a symptomatic large persistent urachal cyst in a patient that had undergone a radical prostatectomy which was complicated by recto-vesical fistula.

## Highlights

• In this case report we present a rare case of a symptomatic urachal cyst requiring surgical resection.
• Postoperative complications after prostatectomy might have contributed to the extensiveness of the condition.
• Histology of the specimen showed mucinous cystadenoma with low malignant potential.

## 1 Introduction

Urachal cysts are congenital anomalies caused by incomplete obliteration of the urachus, the embryonic canal connecting the bladder to the umbilicus. Although mostly asymptomatic and incidentally discovered, these cysts can cause various symptoms, including infection, obstruction, stone formation and, in rare cases, malignancy.^1^^,^^2^ Treatment depends on size, symptoms and the suspicion of malignancy. Surgical resection is usually the treatment of choice.^3^ Localized prostate cancer is treated with robot-assisted radical prostatectomy (RARP). Although urachal cysts are rarely mentioned as a complication during RARP, they can present an unforeseen challenge for both the urologist and the patient. The combination of a urachal cyst and fistula after RARP is noteworthy since the urachus is usually divided during the procedure. The available literature on the best diagnostic and therapeutic approach in this specific context is scarce.^4^

## 2 Case

**Fig. 1:**
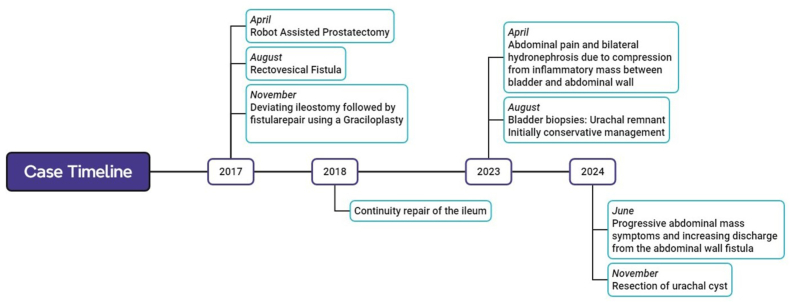
Pre-operative CT scan, sagittal view. The cystic mass measures 20 x 10 × 13 cm.

An 82-year-old male was referred to our university clinic seven years after undergoing a RARP (pT3aNxR1 Gleason 3 + 3 = 6; Fig. 1). The prostatectomy was performed with an anterior approach with transection of the median umbilical ligament (urachus), with no particularities noticed that could indicate a urachal remnant. During the prostatectomy, a rectal lesion of 3 cm occurred which was repaired with a running suture.

During follow-up, he presented with a rectovesical fistula. Initially, he received a laparoscopic deviating ileostomy, followed by a fistula resection with closure of the bladder. A graciloplasty between rectum and urethra was performed. A year later, he received a successful continuity repair of the ileum, and he had no further urinary-intestinal fistula complications. His bladder capacity had deteriorated after these procedures necessitating a condom catheter. A follow-up CT-scan four years later showed no abnormalities.

The patient presented seven years after the initial surgery with abdominal pain and a fistula opening next to the umbilicus with urinary leakage. There was a mass palpable in the lower abdomen, located in the midline between the umbilicus and the bladder. CT imaging showed a 6 × 10 cm mass above the bladder and against the rectum with also hydronephrosis on both sides with minimal contrast perfusion of the right-sided kidney (creatinine clearance 44). Initially a recurrence of prostate cancer was suspected, although his PSA had been stable at 0.07 ng/ml and a PSMA-PET CT was performed with no PSMA activity detected, indicating no significant prostate cancer recurrence or metastasis.

**Fig. 2:**
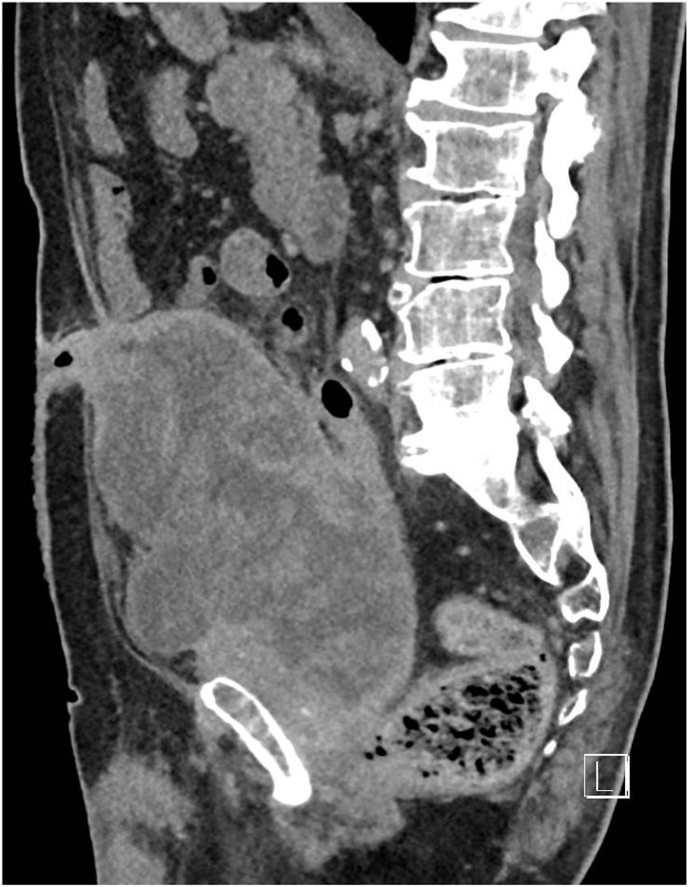
Case timeline. Key clinical events are shown above, with corresponding surgical interventions written below.

Retrospectively, the MRI-scan from 2017 performed during the diagnosis of his prostate cancer did indicate a bladder diverticulum or urachus remnant. Next, a cystoscopy was performed, and histology was obtained from the lesion found in the bladder roof from transurethral biopsies. Pathology showed mucinous epithelium matching a urachal remnant, there were no signs of malignancy. Because of the size of the lesion, age and condition of the patient and limited complaints, a conservative approach was followed. The fistula was flushed daily, and the patient's urine remained clear. However, growth of the lesion was progressive over time and as a result he developed problems with defecation. The mass had now increased to 20x11 × 13 cm (Fig. 2) with the appearance of necrosis within the lesion and compression of the rectum, coecum and terminal ileum on CT imaging. The patient was discussed at a multidisciplinary tumour board meeting with a concurrent assessment by the geriatrician on fitness for surgery and the decision was made to resect the abdominal mass.

**Fig. 3:**
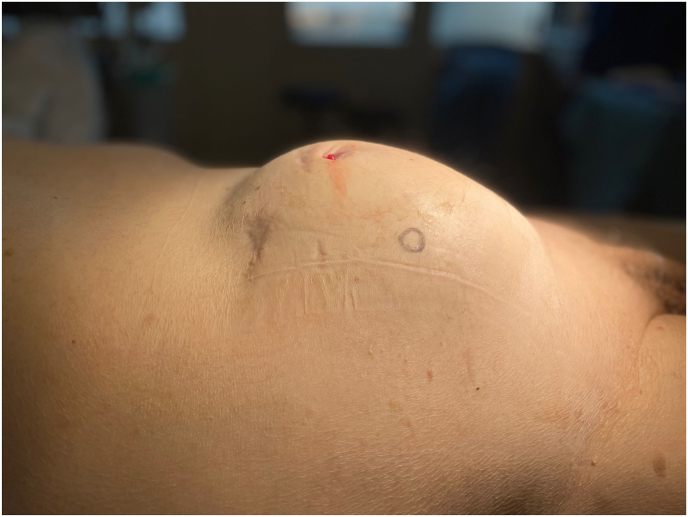
Pre-operative: Abdominal mass visible beneath the skin. Below the umbilicus the fistula opening is visible.

**Fig. 4:**
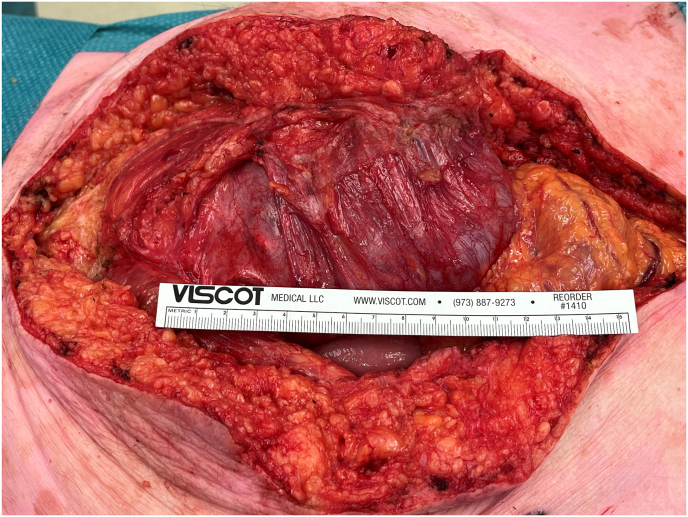
After incision of the abdominal wall the mass was adhesive directly below and with the internal abdominal fascia and peritoneum.

**Fig. 5:**
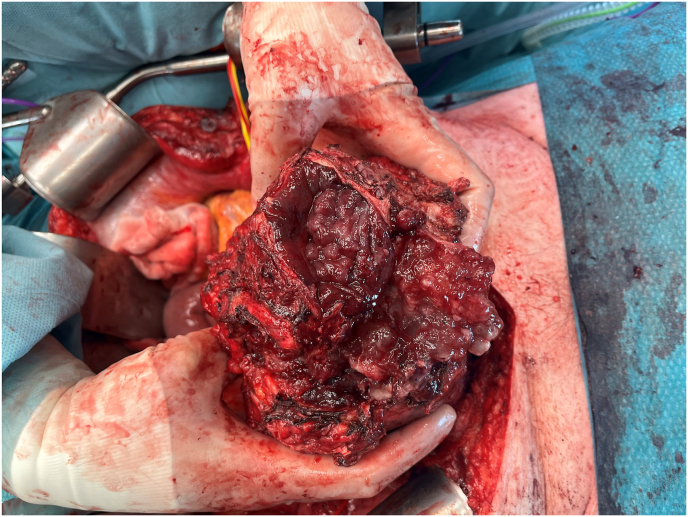
During resection, the necrotic wall ruptured. Mucous tissue was aspirated from within the cyst.

The lesion was surgically approached via median laparotomy (Fig. 3, Fig. 4). There were many intraabdominal adhesions, and the appendix was fused with the mass. An appendectomy was performed. No other fistulae or connections with the bowel were identified. The bladder roof was fused with the urachal mass, and a cystectomy was performed with development of a cutaneous ureterostomy from the remaining functional left kidney. The tumour compressed the iliac vessels bilateral and on the left side encapsulated the iliac vein. This resulted in a partial transection during surgery which required a vascular reconstruction. The entire mass was resected en bloc including the bladder and seminal vesicles (Fig. 5). Postoperatively, the patient developed a thrombus of his reconstructed left iliac external vein for which he was treated with anticoagulants. He also had an ileus which spontaneously improved and a fascial defect of 1.5 cm for which conservative management was sufficient. Finally, the patient could be discharged after 14 days of admission.

**Fig. 6:**
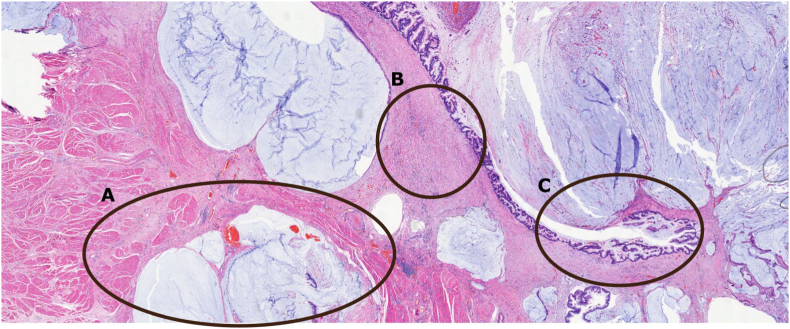
Histology: H&E staining. This slide shows the interface between the cyst and the bladder. At **A:** the detrusor muscle with invasion of mucinous tissue. **B:** extensive inflammation as seen throughout the complete resected specimen. **C:** mucinous epithelium covers the urachal cyst with hyperplastic cells throughout but no evidence of malignancy.

Histology analysis showed no malignancy of the appendix. The cyst was lined with cylindrical mucinous epithelium consistent with a urachal cyst. Mucous deposits were seen in the bladder wall, partially cystic and lined with mucinous epithelium growing into the detrusor without malignant characteristics and created a connection between the larger cyst and the bladder (Fig. 6). Immunohistochemistry staining of CK20 (+), CK7 (+), CDx2 (+) and Beta-catenin (+) was suggestive for a mucinous cystadenoma with borderline malignancy or cystadenoma with low malignant potential. No evidence of carcinoma was identified.

The available literature for cystadenocarcinoma describes invasive growth as a characteristic of carcinoma. However, other reports always mention high-grade dysplastic epithelial groups as invasive. No articles mention mucosal deposits in the bladder wall with a single layer of little dysplastic epithelium, so it remains unclear how to categorise the previously described mucinous deposits and epithelium located in the detrusor and whether it would classify as a carcinoma.^5^

Postoperatively the patient is doing well with a good recovery after surgery. At 6 months follow up there are no complications, and a follow up CT showed no novel abnormalities.

## 3 Discussion

The resected abdominal mass most likely originates from the urachus with histology fitting to mucinous cystadenoma with no carcinoma present. This pathology confirms earlier findings of a urachal remnant on MRI and location of the lesion with a connection to the bladder. This is a surprising diagnosis considering the previous transection of the urachus during surgery and multiple surgical interventions in the lower abdomen and pelvis. Although the mucinous cystadenoma could have originated from the appendix, there were no signs of any pathological abnormalities of the appendix.

Three main histological differential diagnoses were considered: Mucinous cystadenocarcinoma was unlikely in the absence of stromal invasion or cribriform growth patterns. Metastasis from colorectal origin was unlikely given the presence of a cystic stricture, no staining of Beta-catenin in the nuclei and no known primary colorectal malignancy. A benign villous adenoma was considered due to presence of normal epithelium and villi, however the epithelial proliferation and mucinous deposits favoured a mucinous cystadenoma.

It is plausible that the severity of the abnormality was luxated by earlier surgery with possible fistula and adhesions contributing to the extensiveness of the abnormality. In scientific literature on mucinous tumours of the urachus, to date only 57 cases have been described.^6^^,^^7^ None of these had been identified after a radical prostatectomy or any other surgery dissecting the urachus before the case presentation. Of note is one other case were a urachal urothelial carcinoma was identified during a radical prostatectomy^8^. These are incidental findings but about 5 cases presented with symptoms from a mass effect. Another 5 cases presented with mucusuria or haematuria and in 1 case with drainage from the umbilicus comparable to our patient. There are 10 cases of mucinous cystadenoma specifically. None of these were considered malignant and were in 3 cases an incidental finding. In these other cases, patient presented with pain, abdominal mass or urinary symptoms (haematuria or urgency).^9^ Specific considerations in this case were the initially conservative management due to a hostile pelvis from previous surgeries and complications. The surgery proved complicated both due to adhesions and growth around major iliac blood vessels. The 13 patients with mucinous urachal tumours that did have long term follow-up did not reveal local recurrence or distant metastasis.

## 4 Conclusion

Tumours of the Urachus are rare and mostly found incidentally. Symptoms can range from haematuria and obstruction to complaints of metastases such as abdominal pain or weight loss in the case of malignant tumours. This case highlights the need to consider urachal remnants in post-prostatectomy patients presenting with atypical midline masses or fistulas. The delayed presentation may mimic recurrence or a new malignancy. Even though the risk of malignancy is low, symptomatic or complicated lesions such as this case supportsurgical resection.

## CRediT authorship contribution statement

**Anton M.F. Kalsbeek:** Writing – review & editing, Writing – original draft, Visualization, Investigation, Data curation, Conceptualization. **Arnout R. Alberts:** Writing – review & editing, Data curation, Conceptualization. **LuciaL. Rijstenberg:** Writing – review & editing, Data curation, Conceptualization. **John van der Hoeven:** Writing – review & editing, Conceptualization. **Tahlita C.M. Zuiverloon:** Writing – review & editing, Supervision, Project administration, Investigation, Conceptualization.

## Funding

This research did not receive any specific grant from funding agencies in the public, commercial, or not-for-profit sectors.
